# Supercurrent parity meter in a nanowire Cooper pair transistor

**DOI:** 10.1126/sciadv.abm9896

**Published:** 2022-04-22

**Authors:** Ji-Yin Wang, Constantin Schrade, Vukan Levajac, David van Driel, Kongyi Li, Sasa Gazibegovic, Ghada Badawy, Roy L. M. Op het Veld, Joon Sue Lee, Mihir Pendharkar, Connor P. Dempsey, Chris J. Palmstrøm, Erik P. A. M. Bakkers, Liang Fu, Leo P. Kouwenhoven, Jie Shen

**Affiliations:** 1 QuTech and Kavli Institute of NanoScience, Delft University of Technology, 2600 GA Delft, Netherlands.; 2 Department of Physics, Massachusetts Institute of Technology, 77 Massachusetts Avenue, Cambridge, MA 02139, USA.; 3 Beijing National Laboratory for Condensed Matter Physics, Institute of Physics, Chinese Academy of Sciences, Beijing 100190, China.; 4 Department of Applied Physics, Eindhoven University of Technology, 5600 MB Eindhoven, Netherlands.; 5 California NanoSystems Institute, University of California, Santa Barbara, Santa Barbara, CA 93106, USA.; 6 Electrical and Computer Engineering, University of California, Santa Barbara, Santa Barbara, CA 93106, USA.; 7 Materials Department, University of California, Santa Barbara, Santa Barbara, CA 93106, USA.; 8 Microsoft Quantum Lab Delft, 2600 GA Delft, Netherlands.

## Abstract

We study a Cooper pair transistor realized by two Josephson weak links that enclose a superconducting island in an InSb-Al hybrid nanowire. When the nanowire is subject to a magnetic field, isolated subgap levels arise in the superconducting island and, because of the Coulomb blockade, mediate a supercurrent by coherent cotunneling of Cooper pairs. We show that the supercurrent resulting from such cotunneling events exhibits, for low to moderate magnetic fields, a phase offset that discriminates even and odd charge ground states on the superconducting island. Notably, this phase offset persists when a subgap state approaches zero energy and, based on theoretical considerations, permits parity measurements of subgap states by supercurrent interferometry. Such supercurrent parity measurements could, in a series of experiments, provide an alternative approach for manipulating and protecting quantum information stored in the isolated subgap levels of superconducting islands.

## Teaser

A parity-dependent current-phase relation could be measured in an InSb-Al nanowire Cooper pair transistor.

## INTRODUCTION

When two superconducting (SC) leads couple via a Coulomb-blockaded quantum dot (QD), the isolated energy levels on the dot mediate a supercurrent by coherent cotunneling of Cooper pairs (*1*). For the case of a single-level QD, a control knob for the supercurrent direction is given by the charge parity of dot electrons (*1*). Such a parity-controlled supercurrent has been observed in a nanowire (NW) QD Josephson junction (JJ) (*2*, *3*). It is described by the Josephson relation, *I* = (−1)^*n*_0_^*I*_c_ sin (φ), where *I*_c_ is the critical current, φ is the SC phase difference, and *n*_0_ is the number of dot electrons. In general, the Josephson relation can also acquire a phase offset, φ → φ + φ_0_ with φ_0_ ≠ 0, π, when time-reversal symmetry and mirror symmetry are broken (*4*). This breaking occurs, for example, if a spin-orbit coupled QD is subject to a magnetic field (*4*–*7*).

A different possibility of coupling two SC leads is via an SC island with finite charging energy: a “Cooper pair transistor” (CPT) (*8*–*14*). Unlike in the QD JJ, the SC island carries, within its parity lifetime, an even number of electrons in the ground state, as signified by a charging energy that is a 2*e* periodic function of the island gate charge (*e*, elementary charge) (*10*–*12*). In particular, since the odd charge states are energetically unfavorable for a conventional CPT, the Josephson relation is not expected to exhibit a parity-controlled phase offset.

Recently, a CPT has been realized with an indium arsenide–aluminum (Al) hybrid NW (*12*, *13*). In this case, upon increasing a magnetic field parallel to the NW, a transition from a 2*e* periodic switching current to a switching current with even-odd pattern has been observed (*12*). The interpretation is that a low-energy subgap state arises in the SC island, and, depending on its occupancy, the charge ground state carries an even or an odd number of electrons. An open question is if the Josephson relation of an NW CPT exhibits in the presence of subgap states a parity-controlled phase offset.

Here, we address this question with an NW CPT integrated in a superconducting quantum interference device (SQUID). We investigate the previously described situation when the NW CPT is subject to a parallel magnetic field so that subgap levels arise in the SC island and mediate a supercurrent by coherent cotunneling of Cooper pairs. We show that supercurrent resulting from Cooper pair cotunneling exhibits a phase offset, which distinguishes even and odd charge ground states on the SC island. This phase offset persists when a subgap state approaches zero energy and, based on theoretical considerations, may enable parity readout of low-energy subgap states. Such supercurrent parity readout could provide a new approach for manipulating (*15*–*20*) and protecting (*21*, *22*) quantum information stored in the isolated subgap levels of SC islands (*23*–*27*).

## RESULTS

**Fig. 1.**
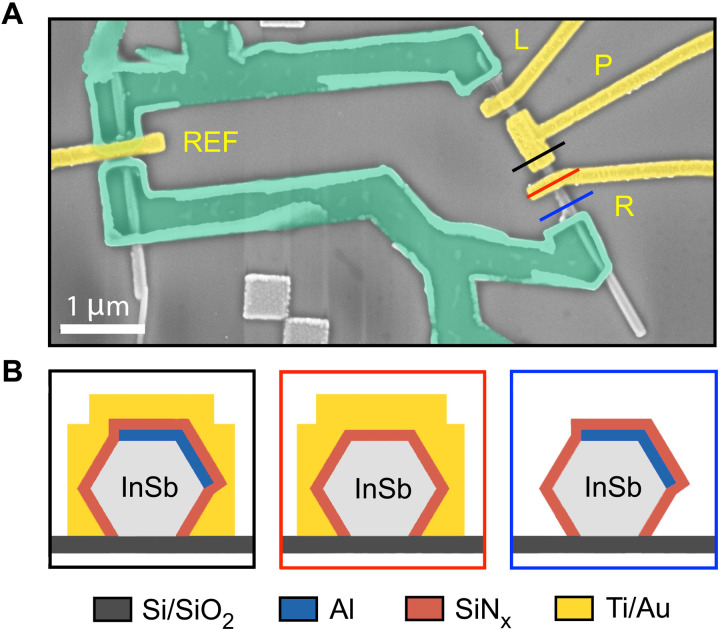
Sketch of the SQUID device. (**A**) False-color micrograph of the measured NbTiN (green) SQUID device comprising an InSb-Al NW CPT in the right arm and an InSb nanowire reference junction in the left arm. Top gates (L, R, and REF) define tunable JJs, and a plunger gate (P) controls the electron number on the hybrid island. The InSb nanowires are **∼**100 nm in diameter, Al shell is **∼**10 nm in thickness, three junctions are **∼**150 nm in length, and the InSb-Al hybrid island is **∼**1 μm in length. (**B**) Cross sections along the lines shown in (A).

The device geometry of our experiment is shown in Fig. 1. For realizing the CPT, we use a shadow-grown (*28*) Al SC island on an indium antimonide (InSb) NW, which couples to two SC Al leads via gate-tunable tunneling barriers. A plunger gate is used for controlling the electron number on the SC island. As we intend to study the full Josephson relation of the NW CPT, we integrate our setup in a SQUID loop made of niobium titanium nitride (NbTiN) and a second InSb NW reference junction. The tunnel coupling of the reference junction is adjustable by a local gate electrode. Concrete fabrication steps are described in Methods.

**Fig. 2.**
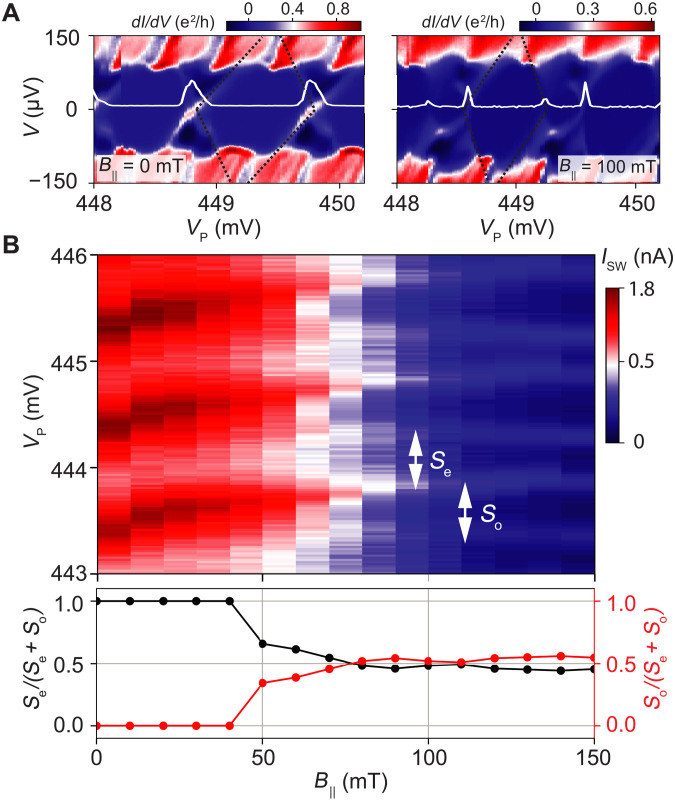
Parity control with magnetic field. (**A**) Differential conductance, *dI*/*dV*, versus source-drain voltage *V* and plunger gate voltage *V*_P_. At zero parallel magnetic field, the differential conductance shows a Coulomb diamond pattern with a 2*e* periodicity. At *B*_||_ = 100 mT, the 2*e* periodicity of the Coulomb diamonds lifts because of the appearance of an odd-parity charge ground state on the SC island. Inset curves show the differential conductance at zero bias. Black dotted lines mark the boundary of a 2*e* charge Coulomb diamond at *B*_||_ = 0 and the boundary of an even-parity Coulomb diamond at *B*_||_ = 100 mT. (**B**) Top: Switching current, *I*_sw_, versus parallel magnetic field *B*_||_ and plunger gate voltage *V*_P_. Bottom: Magnetic field dependence of the normalized even and odd peak spacings, *S*_e_/(*S*_e_ + *S*_o_) and *S*_o_/(*S*_e_ + *S*_o_), showing a transition from a 2*e* periodicity to an even-odd pattern.

Initially, we pinch off the reference junction and characterize the NW CPT by measuring the differential conductance *dI*/*dV* versus the source-drain voltage *V* and the plunger gate voltage *V*_P_. Our results are shown in Fig. 2A for zero and finite parallel magnetic fields *B*_∥_.

At zero magnetic field, we observe a pattern of Coulomb diamonds with sharp edges due to the weak island-lead coupling. Besides the Coulomb diamonds, which signify the importance of charging effects on the SC island, the zero-bias differential conductance exhibits 2*e* periodic oscillations, which implies the transport of Cooper pairs (see the inset curve in Fig. 2A). Furthermore, above an onset voltage *V*_onset_, a 1*e* periodic modulation of the differential conductance appears, which marks the onset of quasiparticle transport. The charging energy, *E*_C_, is estimated to be ∼20 μeV from the 2*e* charge diamond at *B*_∥_ = 0, and the induced gap, Δ_ind_, is extracted to be ∼50 μeV from onset of quasiparticle transport. The relation *E*_C_ < Δ_ind_ is consistent with the condition for 2*e* periodicity of the Coulomb diamonds at zero field (*29*–*31*).

At finite magnetic fields, the aforementioned onset voltage for quasiparticle transport persists. However, below the onset voltage, the Coulomb diamonds split, resulting in an even-odd pattern. We attribute the appearance of this even-odd pattern to low-energy subgap states that form on the SC island. More specifically, the magnetic field induces a Zeeman splitting of spinful, odd-parity states and, thereby, reduces the minimum single-particle excitation energy in the NW CPT. As a result, odd-parity states can detach from the quasiparticle continuum and, because of their enhanced effective *g*-factor in comparison to the Al shell, form isolated levels below the SC gap (*12*, *32*).

Next, we investigate the subgap levels on the SC island in more detail. We lower the island-lead tunneling barriers and, with the reference junction still pinched off, measure the switching current *I*_sw_ as a function of the parallel magnetic field *B*_∥_ and plunger gate voltage *V*_P_. Our results are depicted in Fig. 2B. At zero magnetic field, the switching current exhibits a 2*e* periodic peak spacing implying that the SC island always carries an even number of electrons in its charge ground state (see also fig. S1A). The situation changes upon applying a parallel magnetic field. The magnetic field induces a splitting of the 2*e* periodic peaks, and, as a result, the switching current exhibits a peak spacing with an even-odd pattern (see also fig. S1B). Similar to the differential conductance, we attribute the appearance of this even-odd pattern to charge ground states with even and odd fermion parity on the SC island. Moreover, as shown in Fig. 2B, the extracted peak spacings oscillate as a function of applied magnetic field, as well as the plunger gate voltage, indicating either the anticrossing or the crossing of the lowest-energy subgap state with a second subgap state at higher energy (*30*, *31*).

**Fig. 3.**
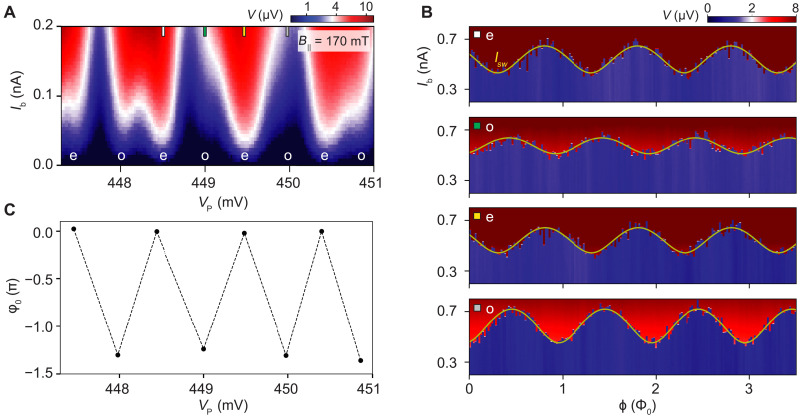
Superconducting phase difference between even and odd parities. (**A**) Voltage drop *V* across the NW CPT versus applied bias current *I*_b_ and plunger gate voltage *V*_P_, showing an even-odd pattern consistent with the appearance of low-energy subgap states at a parallel magnetic field *B*_||_ = 170 mT. (**B**) Voltage drop *V* as a function of the applied bias current *I*_b_ and the flux ϕ that pierces through the SQUID loop for the plunger gate voltages *V*_P_ marked in (A). The fitted switching current *I*_sw_ (yellow) displays a phase offset φ_0_ that discriminates the even and odd charge parity sectors of the SC island. (**C**) Phase offset φ_0_ (relative to the even Coulomb valleys) versus plunger gate voltage *V*_P_. The dashed lines do not represent data but are merely used for improving data visibility. In the range of plunger gate voltages shown here, the phase offset is insensitive.

We now open the reference junction and measure the NW CPT’s full Josephson relation in the presence of low-energy subgap states. For the results presented here, we focus on the magnetic field strength *B*_∥_ = 170 mT and adopt a highly asymmetric SQUID configuration so that the phase drop occurs primarily across the NW CPT. Under these conditions, we apply a bias current *I*_b_ and measure the voltage drop *V* across the SQUID as a function of the plunger gate voltage *V*_P_ and the flux ϕ piercing through the SC loop. Figure 3 shows our measurement data, which we will now discuss in more detail.

Our main finding is that the Josephson relation of the NW CPT exhibits a substantial relative phase offset φ_0_ between Coulomb valleys of opposite charge parity. To determine this phase offset for the Coulomb valleys marked in Fig. 3A, we fit the switching current *I*_sw_ as a function of the flux ϕ. The fitted curves, shown in Fig. 3B, allow us to extract φ_0_ ∼ −1.24π and φ_0_ ∼ −1.31π for the first and second pairs of Coulomb valleys, respectively. For the remaining pairs, we find similar values for the phase offset, as summarized in Fig. 3C. Notably, the leftmost pair of data points in Fig. 3C shows that phase offset persists when the Coulomb peaks are close to a 1*e* spacing (see detailed analysis in fig. S2). Therefore, the phase offset facilitates charge parity readout even if a subgap state is close to zero energy.

**Fig. 4.**
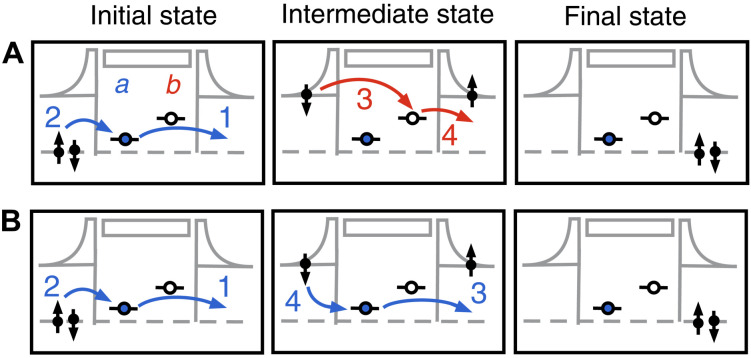
Energy diagrams illustrating Cooper pair transport via subgap levels. (**A**) Typical sequence of intermediate states in which a Cooper pair tunnels between the SC leads (left and right) via the two lowest isolated subgap levels *a* and *b* in the intermediate SC island (center). Such a sequence yields a contribution to the supercurrent proportional to the joint parity of the two subgap levels. In the illustration, numbers indicate the sequence of tunneling events, and solid/empty dots represent filled/empty subgap levels. The occupation numbers of the subgap levels (*n_a_* and *n_b_*) in the sequence are $\left(1,0)\overset{1}{\to }(0,0)\overset{2}{\to }(1,0)\overset{3}{\to }(1,1)\overset{4}{\to }(1,0\right)$. The energy of the initial odd parity (1, 0) configuration is (−1)^*n_a_*+1^ε*_a_* + (−1)^*n_b_*+1^ε*_b_* = ε*_a_* − ε*_b_*, which corresponds to the ground state provided that **ε**_***b***_***>*ε**_***a***_. (**B**) Typical sequence of intermediate states that involves Cooper pair transport via a single subgap level yielding no parity-dependent prefactor. The occupation numbers for this sequence are $\left(1,0)\overset{1}{\to }(0,0)\overset{2}{\to }(1,0)\overset{3}{\to }(0,0)\overset{4}{\to }(1,0\right)$. In (A) and (B), subgap levels are displayed in an “excitation picture” representation (*33*).

Next, we discuss a possible mechanism for a parity-dependent phase offset. We introduce a model for the NW CPT, which comprises a mesoscopic SC island coupled to a pair of *s*-wave SC leads. In our model, we focus on the two lowest isolated subgap levels in the SC island, ±ε*_a_* and ±ε*_b_*, indicated by the peak spacing oscillation as a function of magnetic field and plunger gate in Fig. 2B. Here, we consider two types of cotunneling sequences:

1. In the first type of sequence, shown in Fig. 4A, the Cooper pair splits so that one electron tunnels via ±ε*_a_*, while the other electron tunnels via ±ε*_b_*. For such a two-level sequence, the corresponding supercurrent contribution acquires a prefactor given by the SC island charge parity, (−1)^*n*_0_^. This parity prefactor is analogous to the parity prefactor appearing in the Josephson relation of a QD JJ, where Cooper pairs tunnel via two dot levels with opposite spin polarization (*1*).

2. In the second type of sequence, shown in Fig. 4B, both Cooper pair electrons tunnel via either ±ε*_a_* or ±ε*_b_*. For such a single-level sequence, each of the two electrons contributes a prefactor given by the parity of ±ε*_a_* or ±ε*_b_*. In particular, since the same parity prefactor appears twice in the sequence, it squares to one. Consequently, in the single-level supercurrent contribution, a parity prefactor is absent.

If we collect all sequences, we obtain the Josephson relation (see details in section 4 of the Supplementary Materials)

$$I={\left(-1\right)}^{n_{0}}I_{\text{ab}}\text{sin}\;(\varphi +\varphi_{ab})+\sum_{\ell =a,b}I_{\ell }\text{sin}\;(\varphi +\varphi_{\ell })$$ (1)

Here, *I_ab_* and *I*_ℓ_ are amplitudes, which are 1*e* periodic in the gate charge if the lowest subgap level is at zero energy. Furthermore, the phase offsets φ_ℓ_ arise if the subgap states couple inequivalently to the SC leads (see equation 17 in the Supplementary Materials for the detailed condition on the tunneling amplitudes) and if, due to time-reversal symmetry breaking, the tunnel couplings acquire complex phase factors.

We now highlight two differences between the NW CPT and a QD JJ: First, the island which mediates the Josephson current is in an SC state, not a normal state as for a QD JJ. Consequently, not only conventional tunneling events can occur but also anomalous tunneling events in which an electron is created/destroyed on both the SC island and the leads. Second, for a QD JJ, the wave functions on the dot are highly localized, which justifies a point-like tunneling contact. In comparison, for an NW CPT, the subgap level wave functions can be extended, which induces longer-range island-lead tunnel couplings. In particular, such longer-range couplings can break the mirror symmetry, due to the combined effect of spin-orbit coupling and magnetic field in the tunneling region, and lead to additional contributions to φ*_ab_*, φ_ℓ_.

Returning to Eq. 1, the total phase offset is φ_*n*_0__ ≡ arg [(−1)^*n*_0_^*I_ab_e*^*i*φ*_ab_*^ + ∑_ℓ_*I*_ℓ_*e*^*i*φ_ℓ_^], and the relative phase offset between the parity sectors is φ_0_ ≡ φ_*n*_0_+1_ − φ_*n*_0__. In these expressions, the parity prefactor flips upon tuning the gate charge of the SC island between different charge parity sectors. As a result of these parity flips, the phase offset does not exhibit a 1*e* periodicity in the gate charge even if one of the subgap states is at zero energy. Instead, if *I_ab_* ≠ 0, φ_0_ is always 2*e* periodic and permits the measurement of the parity of the lowest subgap level. To practically enable such parity measurements, the two-level contribution should be sizable, *I_ab_* ≫ *I*_ℓ_. Also, to avoid thermal excitations, the temperature *T* should be small compared to the level separation ∣ε*_a_* − ε*_b_*∣. Therefore interestingly, if ∣ε*_a_* − ε*_b_* ∣ ≳ *T*, the parity prefactor measures the joint parity of ±ε*_a_* and ±ε*_b_*. Such joint parity measurements could be leveraged for entangling qubits stored in the subgap levels of SC islands (*15*–*20*).

**Fig. 5.**
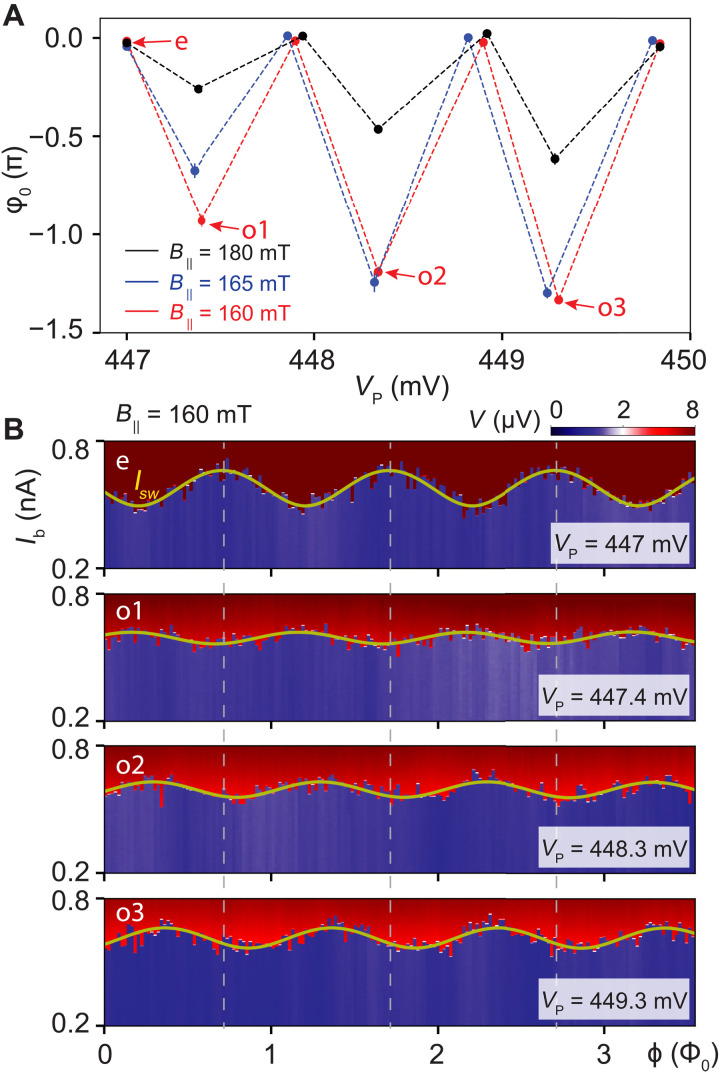
Tunable phase offset. (**A**) Phase offset φ_0_ versus plunger gate voltage *V*_P_ for various parallel magnetic fields *B*_||_. The dashed lines do not represent data but are merely used for improving data visibility. The phase offset is sensitive to both plunger gate voltage and magnetic field variations. (**B**) Voltage drop *V* as a function of the applied bias current *I*_b_ and the SQUID flux ϕ for a parallel magnetic field *B*_||_ = 160 mT. The switching current *I*_sw_ (yellow) displays a phase offset φ_0_ between even (e) and odd (o) Coulomb valleys of the SC island that is tunable by the plunger gate voltage *V*_P_.

So far, we have discussed a regime with substantial φ_0_ for parity readout with maximal resolution. However, such an ideal situation is not always realized. In Fig. 5A, we display the phase offset versus plunger gate voltage for multiple magnetic field values. For a selection of data points, we also show the fitted switching current *I*_sw_ in Fig. 5B. Detailed analysis is shown in figs. S3 to S5. In comparison, there is another regime in which NW CPT exhibits phase independence on its parity (see details in figs. S6 and S7). In Fig. 5, our findings are twofold: First, we observe that the phase offset for subsequent Coulomb valley pairs is tunable by the magnetic field and the plunger gate voltage. Such a tunability arises because both control parameters change the support of the subgap level wave function and, thereby, alter the lead-island Josephson couplings. Second, we find that the phase offset decreases upon increasing the magnetic field. This decrease suggests that the level separation between the lowest-energy and higher-energy subgap states increases so that the supercurrent contribution with the parity-dependent prefactor becomes energetically unfavorable. As a result, in this regime, the NW CPT exhibits a phase dependence that is only weakly dependent on its parity.

## DISCUSSION

In summary, we have studied the Josephson relation of an InSb-Al NW CPT. We have demonstrated that upon applying a magnetic field, subgap levels arise in the SC island and mediate a supercurrent with a parity-dependent phase offset. We have shown that the phase offset persists when the subgap state approaches zero energy and enables parity readout of the lowest energy subgap state. Such a supercurrent parity readout could be useful for the manipulation (*15*–*20*) and protection (*21*, *22*) of qubits stored in the isolated subgap levels of SC islands (*23*–*27*).

## METHODS

### Device fabrication

The InSb NWs used in the experiment were grown on an indium phosphide substrate by metalorganic vapor-phase epitaxy. In the molecular beam epitaxy chamber, Al flux was deposited along a specific direction to form Al shadows on InSb NWs by neighboring NWs (*28*). InSb-Al NWs with shadows were transferred onto a doped Si/SiO*_x_* substrate using a nanomanipulator installed inside a scanning electron microscope. NbTiN superconductor was sputter deposited right after Ar etching dedicated to removing the oxidized layer. Subsequently, 30-nm SiN*_x_* was sputter deposited to work as a dielectric layer, and 10/120-nm Ti/Au was used as a top gate.

### Transport measurement

The sample was measured at a base temperature of ∼20 mK in an Oxford dry dilution refrigerator equipped with a vector magnet. Differential conductance was measured by applying small AC lock-in excitation superimposed on a DC voltage and then measuring AC and DC current through the device. Typically, low frequency of ∼27 Hz and AC excitation amplitude of ∼10 μV were used for lock-in measurement. In current bias measurement, current was applied through the device while monitoring voltage drop on device. The direction of the magnetic field was aligned with respect to the InSb-Al island arm by detecting the supercurrent of CPT while rotating the magnetic field direction.

## Supplementary Materials

### This PDF file includes:

Supplementary Text Figs. S1 to S7 References
