# Supplementary material for: Supercurrent parity meter in a nanowire Cooper pair transistor
Source: Sci Adv. 2022 Apr 22;8(16):eabm9896. doi: 10.1126/sciadv.abm9896 (PMC9032955; doi:10.1126/sciadv.abm9896)
Supplement: Supplementary file 1 — Supplementary Text Figs. S1 to S7 References [file sciadv.abm9896_sm.pdf]

Science Advances
AAAS

Supplementary Materials for

Supercurrent parity meter in a nanowire Cooper pair transistor

Ji-Yin Wang, Constantin Schrade, Vukan Levajac, David van Driel, Kongyi Li, Sasa Gazibegovic, Ghada Badawy, Roy L. M. Op het Veld, Joon Sue Lee, Mihir Pendharkar, Connor P. Dempsey, Chris J. Palmstrøm, Erik P. A. M. Bakkers, Liang Fu, Leo P. Kouwenhoven, Jie Shen*

*Corresponding author. Email: shenjie@iphy.ac.cn

Published 22 April 2022, *Sci. Adv.* **8**, eabm9896 (2022)
DOI: 10.1126/sciadv.abm9896

This PDF file includes:

Supplementary Text
Figs. S1 to S7
References

SECTION1: ADDITIONAL MEASUREMENT DATA

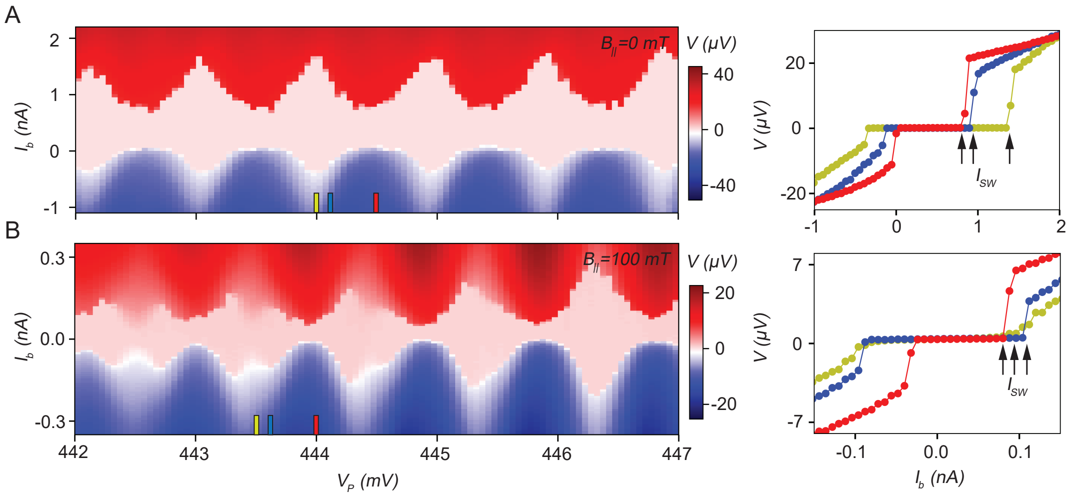

FIG. S1. **Current-bias measurement results at different magnetic fields.** Current-bias characteristics of the NW CPT at different magnetic fields with the reference arm pinched off. (**A**) Left panel: Voltage drop V across the NW CPT as a function of current bias I_b and plunger gate V_P at $B_{||}$=0. Right panel: Linecuts at three different plunger gate values. (**B**) Left panel: Voltage drop V across the NW CPT as a function of I_b and V_P at $B_{||}$=100 mT. Right panel: Linecuts at three different plunger gate values. Black arrows mark the switching current I_{sw}, where the NW CPT transitions from the SC state to the normal state.

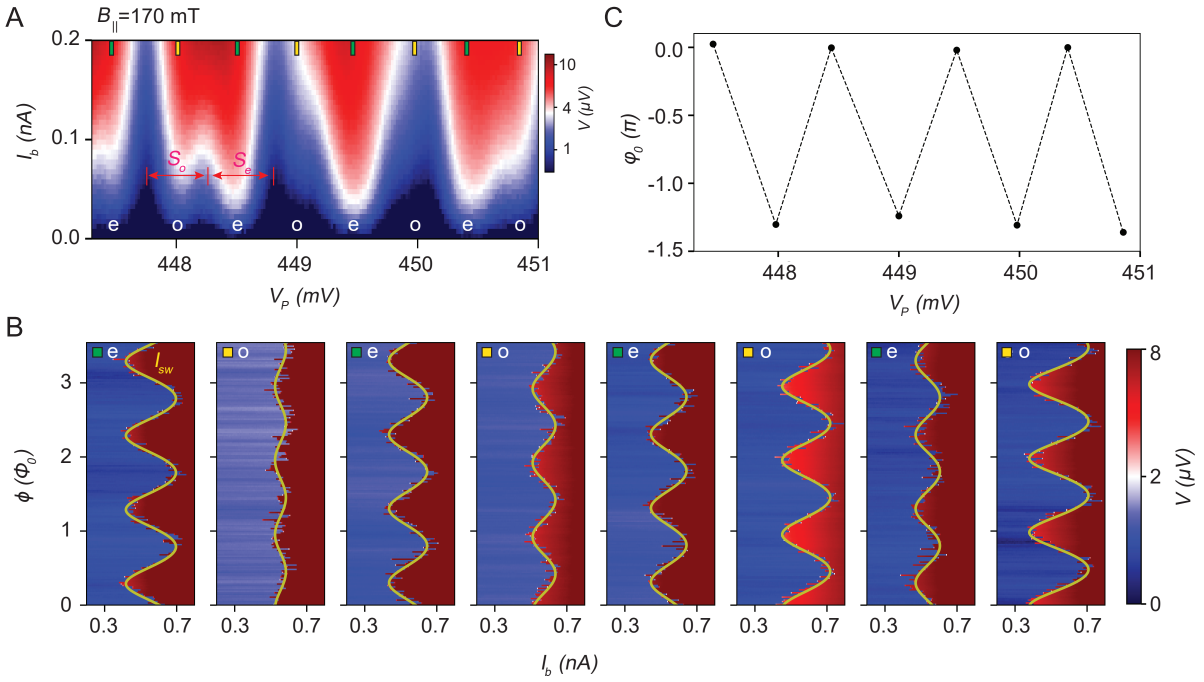

FIG. S2. **Transport characteristics of the NW CPT for even and odd charge parity sectors at $B_{||}$=170 mT.** (**A**) Voltage drop across the NW CPT as a function of current bias I_b and plunger gate V_P with reference arm pinched off. Labels 'e' ('o') indicate Coulomb valleys of even (odd) charge parities of the SC island. Black bars mark the positions of three most left supercurrent peaks and distance between two neighboring peaks gives peak space for even (S_e) or odd parity (S_o). S_e and S_o have comparable values, indicating lowest sub-gap state is close to zero. (**B**) Voltage drop across the SQUID device as a function of current bias I_b and flux ϕ threading SQUID loop at different charge parities of the SC island. The fitted switching current (yellow), I_{sw}, display a phase offset between opposite parity sectors. (**C**) Phase offset φ_0 versus plunger gate V_P. Dashed lines are guide lines to eye. The data shown in this figure was measured after a thermal cycle of the dilution refrigerator, while the data in the subsequent figures was measured before the thermal cycle.

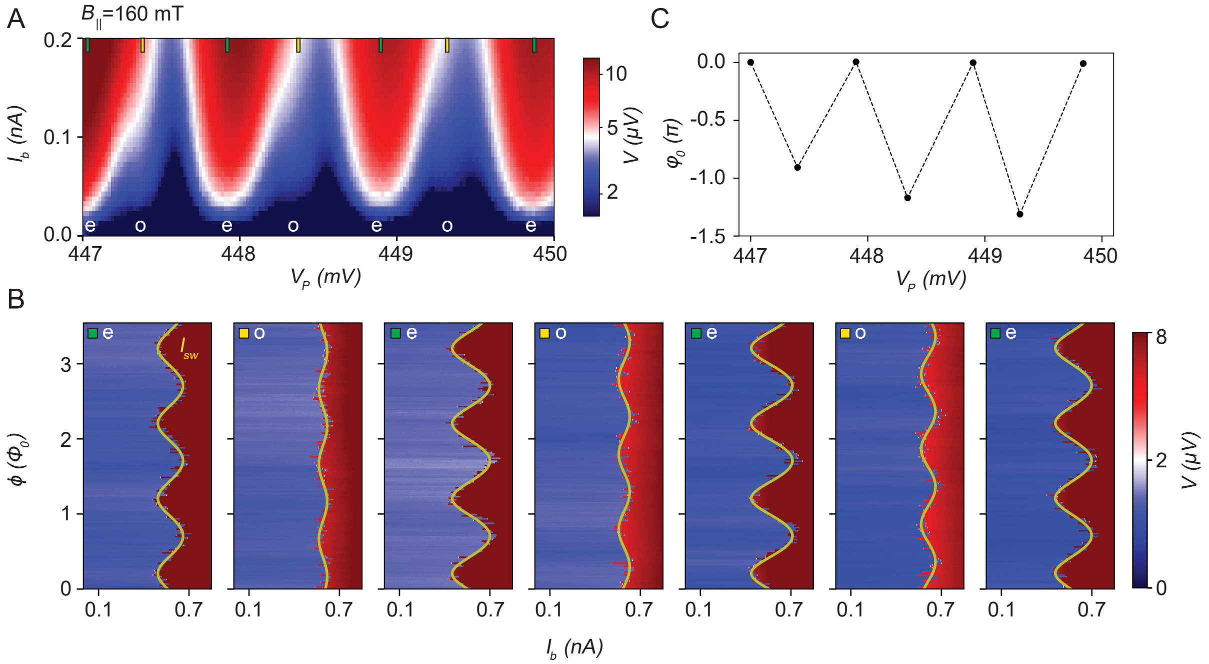

FIG. S3. **Transport characteristics of the NW CPT for even and odd charge parity sectors at $B_{||}$=160 mT.** (**A**) Voltage drop across the NW CPT as a function of current bias I_b and plunger gate V_P with reference arm pinched off. Labels 'e' ('o') indicate Coulomb valleys of even (odd) charge parities of the SC island. (**B**) Voltage drop across the SQUID device as a function of current bias I_b and flux ϕ threading SQUID loop at different charge parities of the SC island. The fitted switching current (yellow), I_{sw}, display a phase offset between opposite parity sectors. (**C**) Phase offset φ_0 versus plunger gate V_P. Dashed lines are guide lines to eye.

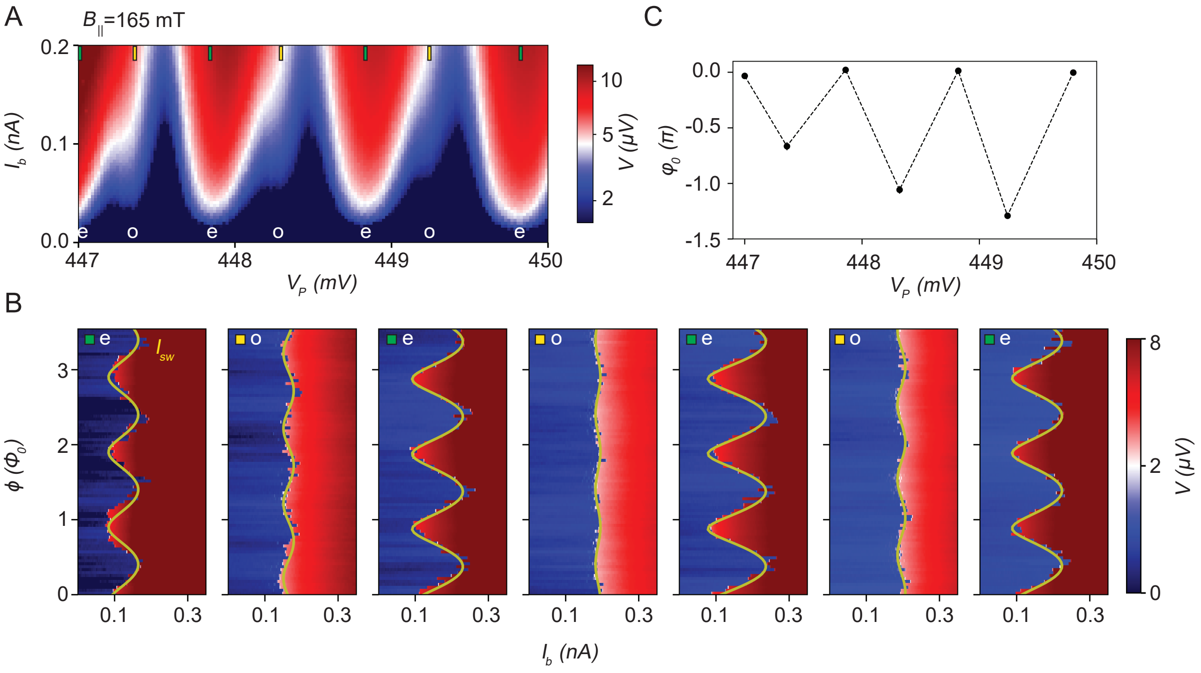

FIG. S4. **Transport characteristics of the NW CPT for even and odd charge parity sectors at $B_{||}$=165 mT.** (**A**) Voltage drop across the NW CPT as a function of current bias I_b and plunger gate V_P with reference arm pinched off. Labels 'e' ('o') indicate Coulomb valleys of even (odd) charge parities of the SC island. (**B**) Voltage drop across the SQUID device as a function of current bias I_b and flux ϕ threading SQUID loop at different charge parities of the SC island. The fitted switching current (yellow), I_{sw}, display a phase offset between opposite parity sectors. (**C**) Phase offset φ_0 versus plunger gate V_P. Dashed lines are guide lines to eye.

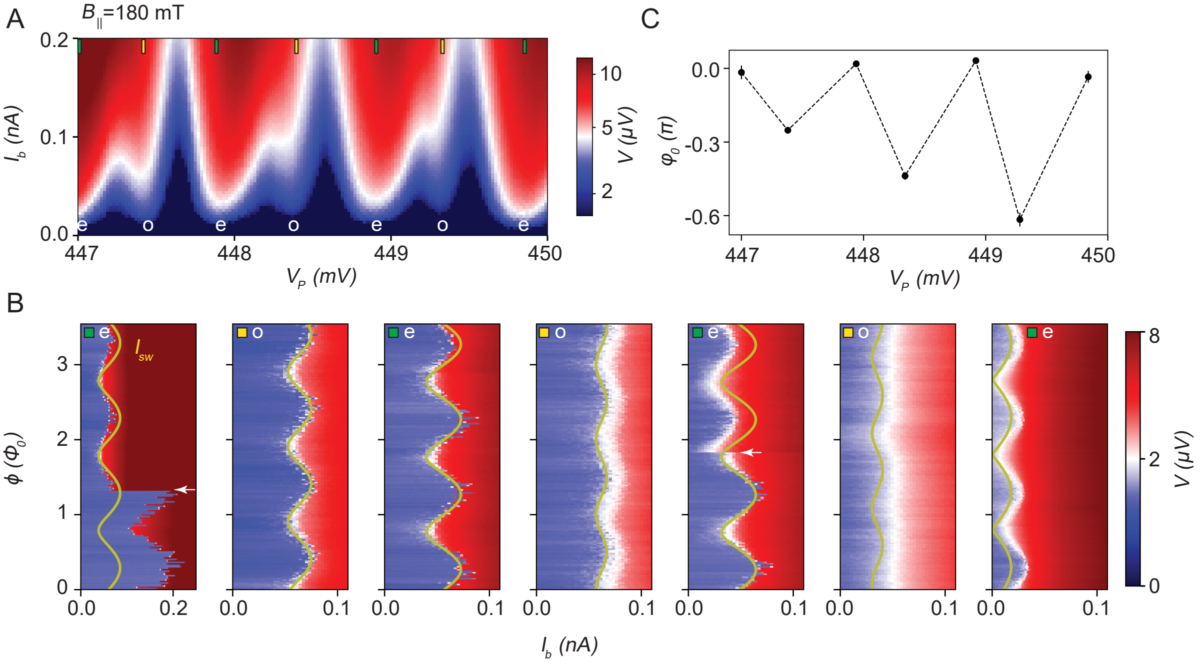

FIG. S5. **Transport characteristics of the NW CPT for even and odd charge parity sectors at $B_{||}$=180 mT.** (**A**) Voltage drop across the NW CPT as a function of current bias I_b and plunger gate V_P with reference arm pinched off. Labels 'e' ('o') indicate Coulomb valleys of even (odd) charge parities of the SC island. (**B**) Voltage drop across the SQUID device as a function of current bias I_b and flux ϕ threading SQUID loop at different charge parities of the SC island. The fitted switching current (yellow), I_{sw}, display a phase offset between opposite parity sectors. White arrows mark where sudden jumps happen, possibly caused by instability of reference gate. In order to reliably fit superconducting phase, the part before or after jump is shifted to align with rest part during fitting. (**C**) Phase offset φ_0 versus plunger gate V_P. Dashed lines are guide lines to eye.

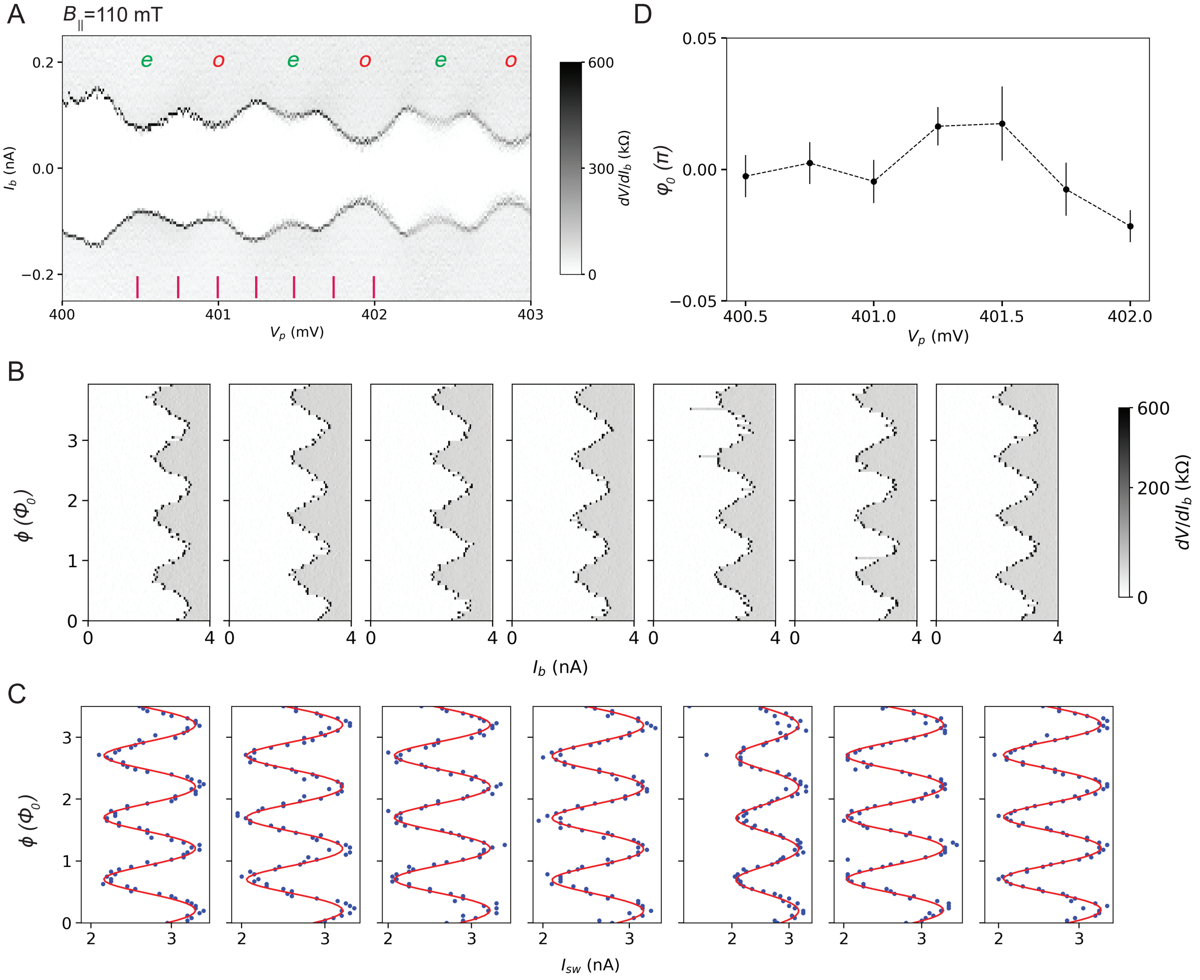

FIG. S6. **A different plunger gate V_P region having phase independence on parities at $B_{||}$=110 mT.** (**A**) Differential resistance of the NW CPT as a function of current bias I_b and plunger gate V_P with reference arm pinched off. Green (red) labels 'e' ('o') indicate Coulomb valleys of even (odd) charge parities of the SC island. (**B**) Differential resistance of the SQUID device as a function of current bias I_b and flux ϕ threading SQUID loop at different plunger gate points marked by red bars in (A). (**C**) Extracted switching current (blue points) versus flux ϕ and corresponding fitting curves (red lines). (**D**) Phase offset φ_0 versus plunger gate V_P. Dashed lines are guide lines to eye. Note that the values φ_0 are obtained by subtrating mean value of all points. The varying of phase with V_P is within error bar fluctuation.

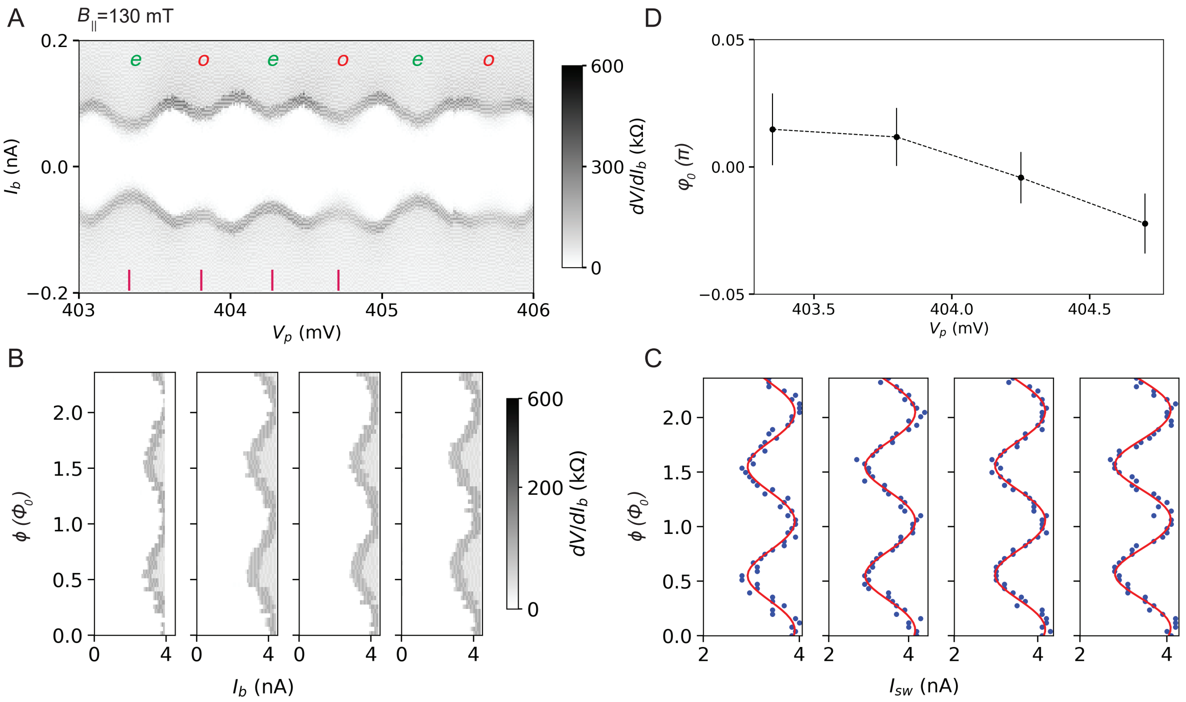

FIG. S7. **Another example of phase independence on parities at $B_{||}$=130 mT.** (**A**) Differential resistance of the NW CPT as a function of current bias I_b and plunger gate V_P with reference arm pinched off. Green (red) labels 'e' ('o') indicate Coulomb valleys of even (odd) charge parities of the SC island. (**B**) Differential resistance of the SQUID device as a function of current bias I_b and flux ϕ threading SQUID loop at different plunger gate points marked by red bars in (A). (**C**) Extracted switching current (blue points) versus flux ϕ and corresponding fitting curves (red lines). (**D**) Phase offset φ_0 versus plunger gate V_P. Dashed lines are guide lines to eye. Note that the values φ_0 are obtained by subtrating mean value of all points. The varying of phase with V_P is within error bar fluctuation. Note that the plunger gate region is very close to that in Fig. S6.

SECTION2: COULOMB VALLEY EXTRACTION

We note that the measured current-voltage I_b-V curves of Cooper-pair transistor exhibit a finite slope for all voltages, see Fig. S2A- S5A. The possible reasons are (1) at finite magnetic field, Josephson energy in the Cooper-pair transistor junctions is suppressed and thermal fluctuation results in resistive electron transport (34, 35); (2) In our device, the leads of Cooper-pair transistor are made from NbTiN/Al and NbTiN is able to push quasiparticle into Al, softening Al gap (36). Coulomb valleys could still be addressed via resistance peak around zero-bias voltage, albiet smeared IV curve resulting from above mentioned two mechanisms, because electron transport is most resistive at Coulomb valleys in both scenarios. Furthermore, both aforementioned mechanisms would not affect superconducting phase measurement results with reference turning on. When reference arm is turned on, total supercurrent (as well as Josephson energy) becomes much larger. Then, thermal fluctuation plays much less of a role and quasi-particle transport is completely suppressed, which is reflected by very sharp transition from superconducting to resistive regime in superconducting phase measurement.

SECTION3: DISCUSSION ON SELF-INDUCTANCE OF SQUID LOOP

According to Tinkham's book (37), a superconducting loop subjected to an external magnetic flux can generate screening current to expel the external flux, which could distort the measured current-phase relationship and make precise extraction of superconducting phase difficult. In order to eliminate the doubt, we quantitatively estimate the amplitude of self-generated flux resulting from inductance. In thin superconducting film, the total inductance, L comprises of kinetic inductance, L_k and geometric inductance, L_g. NbTiN film properties have been systematically studied (38). A typical 100 nm film has a T_c of 14 K, a resistivity of 123 $\mu\Omega$·cm, and $L_k/(L_k{+}L_g)\sim$0.3. In our device, NbTiN has thickness of 80 nm and we adopt the T_c and resistivity from 100 nm film, and $L_k/(L_k{+}L_g)$ is $\sim$ 0.5 by interpolating the data of $L_k/(L_k{+}L_g)$ versus film thickness. Kinetic inductance L_k can be calculated from Eq. (6) in reference (39). We suppose that the critical current I_c of the loop is 10 nA (actual measured switching current of Cooper-pair transistor is alway below 2 nA in our measurement), and the value of $I_c{\cdot}L \sim 4\times10^{-4}\Phi_0$, where Φ_0 is flux quantum. Thus, self-inductance is negligibly small compared with external flux and was not taken into account during data analysis.

SECTION4: EFFECTIVE HAMILTONIAN FOR THE NANOWIRE COOPER-PAIR TRANSISTOR

In this section of the Supplemental Material, we present more details on the derivation of the effective Hamiltonian for the NW CPT which yields a Josephson relation with parity-dependent phase offset. We proceed in multiple steps:

Step 1: Model Hamiltonian

As a first step, we introduce our model Hamiltonian, which comprises a SC island with subgap states coupled to a pair of s-wave SC leads. The Hamiltonian for the SC leads has the form,

$$H_{\rm SC} = \sum_{\ell={\rm L,R}} \sum_{\mathbf{k}} \Psi^\dagger_{\ell,\mathbf{k}} \left(\xi_{\mathbf{k}}\eta_z + \Delta_\ell \eta_x e^{i\varphi_\ell \eta_z}\right) \Psi_{\ell,\mathbf{k}}, \tag{1}$$

where $\Psi_{\ell,\mathbf{k}} = (c_{\ell,\mathbf{k}\uparrow}, c^\dagger_{\ell,-\mathbf{k}\downarrow})^T$ denotes the Nambu spinor with the electron annihilation operator $c_{\ell,\mathbf{k}s}$ for momentum $\mathbf{k}$, spin s, and lead ℓ. Furthermore, $\eta_{x,y,z}$ are the Nambu-space Pauli matrices, and $\xi_{\mathbf{k}}$ is the normal state dispersion. The magnitudes and the phases of the SC order parameters are Δ_ℓ and φ_ℓ, respectively. For simplicity, we assume that $\Delta_1 = \Delta_2 \equiv \Delta$.

Next, we introduce the charging Hamiltonian for the mesoscopic SC island,

$$U_{\rm C}(n) = U\left(n - n_g\right)^2, \tag{2}$$

where U denotes the charging energy magnitude. Moreover, n is the number operator that counts the electron charges on the island, and n_g denotes the induced charge, which is continuously tunable through the plunger gate voltage. As outlined in the main text, we focus on the two lowest energy subgap levels in the SC island, which will mediate

the Josephson current between the SC leads. In terms of Majorana operators, $\gamma_i = \gamma_i^\dagger$, the Hamiltonian for the two subgap states reads,

$$H_{\rm SG} = i\varepsilon_a\,\gamma_{1a}\gamma_{2a} + i\varepsilon_b\,\gamma_{1b}\gamma_{2b}, \tag{3}$$

where $\varepsilon_{a,b}$ are the energy splittings. We adjust the induced charge so that the SC island hosts n_0 electron charges in its ground state and, as a result, the joint fermion parity of the subgap levels satisfies,

$$\gamma_{1a}\gamma_{2a}\gamma_{1b}\gamma_{2b} = (-1)^{n_0}. \tag{4}$$

Lastly, we introduce the tunneling Hamiltonian to describe the coupling between the SC leads and the SC island,

$$H_{\rm T} = \sum_{\ell,i}\sum_{\mathbf{k}s}\lambda^s_{\ell i}\;c^\dagger_{\ell,\mathbf{k}s}\gamma_i e^{-i\phi/2} + \text{H.c.} \tag{5}$$

Here, $\lambda^s_{\ell i}$ are complex tunneling amplitudes which connect electrons on the SC lead ℓ to the subgap states, which are described by the Majorana operators γ_i. Furthermore, $e^{\pm i\phi/2}$ increases/decreases the number of electrons on the SC island by one unit, $[n, e^{\pm i\phi/2}] = \pm e^{\pm i\phi/2}$, while the Majorana operators γ_i induce flips of the SC island parity. In summary, the total Hamiltonian for our model is given by,

$$H = H_{\rm SC} + U_{\rm C} + H_{\rm SG} + H_{\rm T}. \tag{6}$$

Step 2: Effective Hamiltonian (General form)

As a second step, we provide an overview of the effective Hamiltonian for the model that we introduced in the previous subsection. More specifically, up to fourth order in the tunnel couplings $\lambda^s_{\ell i}$, the effective Hamiltonian reads,

$$H_{\rm eff} = P\,H_{\rm T}\left\{[u(n_a,n_b) - H_{\rm SC} - U_{\rm C} - H_{\rm SG}]^{-1}(1-P)H_{\rm T}\right\}^3 P. \tag{7}$$

Here, P denotes the projection operator on the subspace of $H_{\rm SC} + H_{\rm C} + H_{\rm SG} + H_{\rm T}$ with a fixed charge configuration (n_a, n_b) at energy,

$$u(n_a,n_b) = U_{\rm C}\,(n_a + n_b - n_g)^2 + (-1)^{n_a+1}\varepsilon_a + (-1)^{n_b+1}\varepsilon_b. \tag{8}$$

To evaluate $H_{\rm eff}$ based on the equation presented above, we need to compute all sequences of intermediate states that mediate a Cooper pair between the SC leads via the SC island. Before going into the details of this calculation, we first present the general result,

$$\begin{aligned} H_{\rm eff} &= -\gamma_{1a}\gamma_{2a}\gamma_{1b}\gamma_{2b}\left[\beta\sum_{m=1}^{4}J^{(m)}_{ab}\cos(\varphi+\varphi^{(m)}_{ab})\right] - \alpha_a J_a\cos(\varphi+\varphi_a) - \alpha_b J_b\cos(\varphi+\varphi_b) \\ &\equiv -\gamma_{1a}\gamma_{2a}\gamma_{1b}\gamma_{2b}\,\beta\,J_{ab}\cos(\varphi+\varphi_{ab}) - \alpha_a J_a\cos(\varphi+\varphi_a) - \alpha_b J_b\cos(\varphi+\varphi_b) \end{aligned} \tag{9}$$

In the second line, we have defined the Josephson couplings and phase offsets,

$$J_{ab} = \left|\sum_{m=1}^{4}J^{(m)}_{ab}e^{i\varphi^{(m)}_{ab}}\right|, \qquad \varphi_{ab} = \arg\left(\sum_{m=1}^{4}J^{(m)}_{ab}e^{i\varphi^{(m)}_{ab}}\right). \tag{10}$$

We present expressions for the Josephson couplings $(J_a, J_b, J^{(m)}_{ab})$, the phase offsets $(\varphi_a, \varphi_b, \varphi^{(m)}_{ab})$, as well as the dimensionless functions $(\alpha_a, \alpha_b, \beta)$ in the subsequent sections. Here, we only note that for a fixed charge configuration (n_a, n_b), the Josephson relation of the NW CPT is given by,

$$I = (-1)^{n_0}\,I_{ab}\cos(\varphi+\varphi_{ab}) + I_a\cos(\varphi+\varphi_a) + I_b\cos(\varphi+\varphi_b), \tag{11}$$

where $I_{ab} = 2e\beta\,J_{ab}/\hbar$, $I_a = 2e\alpha_a\,J_a/\hbar$, and $I_b = 2e\alpha_b\,J_b/\hbar$. This is the result presented in Eq. 1 of the main text.

Step 3: Effective Hamiltonian (Josephson couplings)

As a third step, we give the expressions for the Josephson couplings ($J_a, J_b, J_{ab}^{(m)}$) as well as the dimensionless functions ($\alpha_a, \alpha_b, \beta$), which appear in the previously introduced effective Hamiltonian H_{eff}.

We, initially, define the auxiliary Josephson couplings,

$$J_{k,\ell}^{i,j} = -\frac{8}{\pi^2\Delta}\left(\sqrt{\Gamma_{\text{L}i}^{\downarrow}\Gamma_{\text{L}j}^{\uparrow}} - \sqrt{\Gamma_{\text{L}j}^{\downarrow}\Gamma_{\text{L}i}^{\uparrow}}\right)\left(\sqrt{\Gamma_{\text{R}k}^{\downarrow}\Gamma_{\text{R}\ell}^{\uparrow}} - \sqrt{\Gamma_{\text{R}\ell}^{\downarrow}\Gamma_{\text{R}k}^{\uparrow}}\right), \tag{12}$$

together with the linewidths,

$$\begin{aligned} \Gamma_{m,1a}^{s} &= \pi\nu_F|\lambda_{m,1a}^{s}|^2 \quad , \quad \Gamma_{m,2a}^{s} = \pi\nu_F|\lambda_{m,2a}^{s}|^2 \\ \Gamma_{m,1b}^{s} &= \pi\nu_F|\lambda_{m,1b}^{s}|^2 \quad , \quad \Gamma_{m,2b}^{s} = \pi\nu_F|\lambda_{m,2b}^{s}|^2. \end{aligned} \tag{13}$$

Here, for example, $\Gamma_{m,1a}^{s}$ denotes the linewidth that the level a acquires due to the tunneling of electrons with spin s from lead m into γ_{1a}. The normal-state density of states at the Fermi level for the leads is given by ν_F. If, we assume an approximate linewidth $\Gamma^{\text{approx}} = 0.01\,\text{meV}$ and a SC gap $\Delta = 0.3\,\text{meV}$ in the SC leads, we find an upper-bound estimate for the critical current $I_c^{\text{approx}} = [16e(\Gamma^{\text{approx}})^2]/(\hbar\pi^2\Delta) \approx 0.1\,\text{nA}$, which is consistent with the values measured in the experiment.

With these definitions, the Josephson couplings, appearing in H_{eff}, are given by,

$$\begin{aligned} J_a &= J_{1a,2a}^{1a,2a} \qquad J_b = J_{1b,2b}^{1b,2b} \\ J_{ab}^{(1)} &= J_{2a,2b}^{1a,1b} \quad , \quad J_{ab}^{(2)} = J_{1a,2b}^{1b,2a} \quad , \quad J_{ab}^{(3)} = J_{1b,2a}^{1a,2b} \quad , \quad J_{ab}^{(4)} = J_{1a,1b}^{2a,2b}. \end{aligned} \tag{14}$$

For introducing the dimensionless functions ($\alpha_a, \alpha_b, \beta$), we first define,

$$g(x) = \sqrt{1+x^2} > 0 \quad , \quad h(n_a+N_a, n_b+N_b) = \frac{u(n_a+N_a, n_b+N_b) - u(n_a,n_b)}{\Delta} > 0, \tag{15}$$

which allows us to write,

$$\begin{aligned}
\alpha_a &= \int_1^\infty \mathrm{d}x \int_1^\infty \mathrm{d}y \, \frac{1}{g(x)g(y)[g(x)+h(n_a-1,n_b)][g(x)+g(y)][g(x)+h(n_a+1,n_b)]}, \\
\alpha_b &= \int_1^\infty \mathrm{d}x \int_1^\infty \mathrm{d}y \, \frac{1}{g(x)g(y)[g(x)+h(n_a,n_b-1)][g(x)+g(y)][g(x)+h(n_a,n_b+1)]}, \\
\beta_1 &= \frac{1}{4}\int_1^\infty \mathrm{d}x \int_1^\infty \mathrm{d}y \, \frac{1}{g(x)g(y)[g(y)+h(n_a,n_b+1)][g(x)+g(y)][g(x)+h(n_a+1,n_b)]} \\
\beta_2 &= \frac{1}{4}\int_1^\infty \mathrm{d}x \int_1^\infty \mathrm{d}y \, \frac{1}{g(x)g(y)[g(x)+h(n_a,n_b-1)][g(x)+g(y)][g(x)+h(n_a+1,n_b)]} \\
\beta_3 &= \frac{1}{4}\int_1^\infty \mathrm{d}x \int_1^\infty \mathrm{d}y \, \frac{1}{g(x)g(y)[g(y)+h(n_a+1,n_b)][g(x)+g(y)+h(n_a+1,n_b-1)][g(x)+h(n_a+1,n_b)]} \\
\beta_4 &= \frac{1}{4}\int_1^\infty \mathrm{d}x \int_1^\infty \mathrm{d}y \, \frac{1}{g(x)g(y)[g(x)+h(n_a,n_b-1)][g(x)+g(y)+h(n_a+1,n_b-1)][g(x)+h(n_a+1,n_b)]} \\
\beta_5 &= \frac{1}{4}\int_1^\infty \mathrm{d}x \int_1^\infty \mathrm{d}y \, \frac{1}{g(x)g(y)[g(x)+h(n_a-1,n_b)][g(x)+g(y)+h(n_a-1,n_b+1)][g(y)+h(n_a-1,n_b)]} \\
\beta_6 &= \frac{1}{4}\int_1^\infty \mathrm{d}x \int_1^\infty \mathrm{d}y \, \frac{1}{g(x)g(y)[g(y)+h(n_a,n_b+1)][g(x)+g(y)+h(n_a-1,n_b+1)][g(y)+h(n_a-1,n_b)]} \\
\beta_7 &= \frac{1}{4}\int_1^\infty \mathrm{d}x \int_1^\infty \mathrm{d}y \, \frac{1}{g(x)g(y)[g(x)+h(n_a,n_b-1)][g(x)+g(y)][g(y)+h(n_a-1,n_b)]} \\
\beta_8 &= \frac{1}{4}\int_1^\infty \mathrm{d}x \int_1^\infty \mathrm{d}y \, \frac{1}{g(x)g(y)[g(y)+h(n_a,n_b+1)][g(x)+g(y)][g(y)+h(n_a-1,n_b)]} \\
\beta &= \sum_{p=1}^{16} \beta_p.
\end{aligned} \tag{16}$$

In the definition of β, we have included parameters $\beta_9, \dots, \beta_{16}$ which are identical to $\beta_1, \dots, \beta_8$ but with the arguments of h interchanged, $h(n,m) \to h(m,n)$. We note that for a substantial charging energy, virtual states with two additional electrons on the SC island are energetically unfavorable and, for simplicity, have not been accounted for in the expressions for $(\alpha_a, \alpha_b, \beta)$.

Step 4: Effective Hamiltonian (Anomalous phase shifts)

As a fourth step, we give the expressions for the phase offsets $(\varphi_a, \varphi_b, \varphi_{ab}^{(m)})$ appearing in the effective Hamiltonian $H_{\rm eff}$.

We, therefore, introduce the auxiliary phase offsets,

$$\varphi_{k,\ell}^{i,j} = \arg[(\lambda_{\mathrm{L}i}^{\downarrow}\lambda_{\mathrm{L}j}^{\uparrow} - \lambda_{\mathrm{L}i}^{\uparrow}\lambda_{\mathrm{L}j}^{\downarrow})^*(\lambda_{\mathrm{R}k}^{\downarrow}\lambda_{\mathrm{R}\ell}^{\uparrow} - \lambda_{\mathrm{R}k}^{\uparrow}\lambda_{\mathrm{R}\ell}^{\downarrow})], \tag{17}$$

which allow us express the phase offsets appearing in $H_{\rm eff}$ as,

$$\begin{aligned} &\varphi_a = \varphi_{1a,2a}^{1a,2a} \quad , \quad \varphi_b = \varphi_{1b,2b}^{1b,2b} \\ &\varphi_{ab}^{(1)} = \varphi_{2a,2b}^{1a,1b} \quad , \quad \varphi_{ab}^{(2)} = \varphi_{1a,2b}^{1b,2a} \quad , \quad \varphi_{ab}^{(3)} = \varphi_{1b,2a}^{1a,2b} \quad , \quad \varphi_{ab}^{(4)} = \varphi_{1a,1b}^{2a,2b}. \end{aligned} \tag{18}$$

Step 5: Effective Hamiltonian (Example calculation)

As a final step, we provide specific examples on sequences of intermediate states that mediate a contribution to the Josephson current with and without a parity-dependent prefactor. We, thereby, focus on the sequences which we have shown in Fig. 4 of the main text.

We begin by considering sequences of the type shown in Fig. 4A, which comprise a parity-dependent prefactor. An example, for such a sequence is given by,

$$\begin{aligned} &P(c_{\mathrm{R},-\mathbf{q}\downarrow}^{\dagger}\gamma_{2,b}e^{-i\phi/2})(\gamma_{1,b}c_{\mathrm{L},-\mathbf{k}\downarrow}e^{i\phi/2})(\gamma_{1,a}c_{\mathrm{L},\mathbf{k}\uparrow}e^{i\phi/2})(c_{\mathrm{R},\mathbf{q}\uparrow}^{\dagger}\gamma_{2,a}e^{-i\phi/2})P \\ &= P(c_{\mathrm{R},-\mathbf{q}\downarrow}^{\dagger}\gamma_{2,b}\gamma_{1,b}c_{\mathrm{L},-\mathbf{k}\downarrow}\gamma_{1,a}c_{\mathrm{L},\mathbf{k}\uparrow}c_{\mathrm{R},\mathbf{q}\uparrow}^{\dagger}\gamma_{2,a})P \\ &= -P(\gamma_{1,a}\gamma_{2,a}\gamma_{1,b}\gamma_{2,b})(c_{\mathrm{R},-\mathbf{q}\downarrow}^{\dagger}c_{\mathrm{L},-\mathbf{k}\downarrow}c_{\mathrm{L},\mathbf{k}\uparrow}c_{\mathrm{R},\mathbf{q}\uparrow}^{\dagger})P \\ &= e^{i(\varphi_{\mathrm{L}}-\varphi_{\mathrm{R}})}\, u_{\mathbf{q}}v_{\mathbf{q}}u_{\mathbf{k}}v_{\mathbf{k}}\, P(\gamma_{1,a}\gamma_{2,a}\gamma_{1,b}\gamma_{2,b})(\gamma_{\mathrm{R},\mathbf{q}\uparrow}\gamma_{\mathrm{L},-\mathbf{k}\downarrow}\gamma_{\mathrm{L},-\mathbf{k}\downarrow}^{\dagger}\gamma_{\mathrm{R},\mathbf{q}\uparrow}^{\dagger})P \\ &= e^{i(\varphi_{\mathrm{L}}-\varphi_{\mathrm{R}})}\, u_{\mathbf{q}}v_{\mathbf{q}}u_{\mathbf{k}}v_{\mathbf{k}}\, P(\gamma_{1,a}\gamma_{2,a}\gamma_{1,b}\gamma_{2,b})P \end{aligned} \tag{19}$$

In the third equality, we have represented the electron operators in the SC leads in terms of Bogoliubov quasiparticles through the relations, $c_{\ell,\mathbf{k}\uparrow} = e^{i\varphi_\ell/2}(u_{\mathbf{k}}\gamma_{\ell,\mathbf{k}\uparrow} + v_{\mathbf{k}}\gamma_{\ell,-\mathbf{k}\downarrow}^{\dagger})$ and $c_{\ell,-\mathbf{k}\downarrow} = e^{i\varphi_\ell/2}(u_{\mathbf{k}}\gamma_{\ell,-\mathbf{k}\downarrow} - v_{\mathbf{k}}\gamma_{\ell,\mathbf{k}\uparrow}^{\dagger})$ with the coherence factors $u_{\mathbf{k}}$, $v_{\mathbf{k}}$. If we sum over all momenta, we find that the amplitude for the example sequence is given by,

$$-(\lambda_{\mathrm{L},1a}^{\uparrow}\lambda_{\mathrm{L},1b}^{\downarrow})^*(\lambda_{\mathrm{R},2a}^{\uparrow}\lambda_{\mathrm{R},2b}^{\downarrow})\sum_{\mathbf{k},\mathbf{q}}\frac{v_{\mathbf{q}}u_{\mathbf{k}}u_{\mathbf{q}}v_{\mathbf{k}}}{[E_{\mathbf{q}}+u(n_a,n_b+1)-u(n_a,n_b)][E_{\mathbf{k}}+E_{\mathbf{q}}][E_{\mathbf{q}}+u(n_a-1,n_b)-u(n_a,n_b)]}, \tag{20}$$

where the $E_{\mathbf{k}} = \sqrt{\xi_{\mathbf{k}}^2 + \Delta^2}$ denotes the dispersion of the SC leads. If we assume a constant density of states ν_F at the Fermi level, we can rewrite this amplitude as,

$$-\frac{1}{\Delta}\int_1^\infty \mathrm{d}x \int_1^\infty \mathrm{d}y\, \frac{\nu_F^2(\lambda_{\mathrm{L},1a}^{\uparrow}\lambda_{\mathrm{L},1b}^{\downarrow})^*(\lambda_{\mathrm{R},2a}^{\uparrow}\lambda_{\mathrm{R},2b}^{\downarrow})}{g(x)g(y)[g(y)+h(n_a,n_b+1)][g(x)+g(y)][g(y)+h(n_a-1,n_b)]}. \tag{21}$$

Hence, we conclude that the sequence contributes to the term $\propto \beta_8$ in the Josephson relation of the NW CPT.

Next, we consider sequences of the type shown in Fig. 4B, which do not comprise a parity-dependent prefactor. An example, for such a sequence is given by,

$$\begin{aligned} & P(\gamma_{1,a}c_{\mathrm{L},-\mathbf{k}\downarrow}e^{i\phi/2})(c^\dagger_{\mathrm{R},-\mathbf{q}\downarrow}\gamma_{2,a}e^{-i\phi/2})(c^\dagger_{\mathrm{R},\mathbf{q}\uparrow}\gamma_{2,a}e^{-i\phi/2})(\gamma_{1,a}c_{\mathrm{L},\mathbf{k}\uparrow}e^{i\phi/2})P \\ &= P(\gamma_{1,a}c_{\mathrm{L},-\mathbf{k}\downarrow}c^\dagger_{\mathrm{R},-\mathbf{q}\downarrow}\gamma_{2,a}c^\dagger_{\mathrm{R},\mathbf{q}\uparrow}\gamma_{2,a}\gamma_{1,a}c_{\mathrm{L},\mathbf{k}\uparrow})P \\ &= P(c_{\mathrm{L},-\mathbf{k}\downarrow}c^\dagger_{\mathrm{R},-\mathbf{q}\downarrow}c^\dagger_{\mathrm{R},\mathbf{q}\uparrow}c_{\mathrm{L},\mathbf{k}\uparrow})P \\ &= -e^{i(\varphi_\mathrm{L}-\varphi_\mathrm{R})}\, u_\mathbf{q}v_\mathbf{q}u_\mathbf{k}v_\mathbf{k}\, P(\gamma_{\mathrm{L},-\mathbf{k}\downarrow}\gamma_{\mathrm{R},\mathbf{q}\uparrow}\gamma^\dagger_{\mathrm{R},\mathbf{q}\uparrow}\gamma^\dagger_{\mathrm{L},-\mathbf{k}\downarrow})P \\ &= -e^{i(\varphi_\mathrm{L}-\varphi_\mathrm{R})}\, u_\mathbf{q}v_\mathbf{q}u_\mathbf{k}v_\mathbf{k} \end{aligned} \tag{22}$$

If we again sum over all momenta, we find that the amplitude for the example sequence is given by,

$$(\lambda^\uparrow_{\mathrm{L},1a}\lambda^\downarrow_{\mathrm{L},1a})^*(\lambda^\uparrow_{\mathrm{R},2a}\lambda^\downarrow_{\mathrm{R},2a})\sum_{\mathbf{k},\mathbf{q}}\frac{v_\mathbf{q}u_\mathbf{k}u_\mathbf{q}v_\mathbf{k}}{[E_\mathbf{k}+u(n_a-1,n_b)-u(n_a,n_b)][E_\mathbf{k}+E_\mathbf{q}][E_\mathbf{k}+u(n_a+1,n_b)-u(n_a,n_b)]}. \tag{23}$$

In particular, if we assume constant density of states ν_F at the Fermi level, we can rewrite this amplitude as,

$$\frac{1}{\Delta}\int_1^\infty \mathrm{d}x \int_1^\infty \mathrm{d}y\, \frac{\nu_F^2(\lambda^\uparrow_{\mathrm{L},1a}\lambda^\downarrow_{\mathrm{L},1a})^*(\lambda^\uparrow_{\mathrm{R},2a}\lambda^\downarrow_{\mathrm{R},2a})}{g(x)g(y)[g(x)+h(n_a-1,n_b)][g(x)+g(y)][g(x)+h(n_a+1,n_b)]}. \tag{24}$$

We, thus, conclude that the sequence contributes to the term $\propto \alpha_a$ in the Josephson relation of the NW CPT.

REFERENCES AND NOTES

1. B. I. Spivak, S. A. Kivelson, Negative local superfluid densities: The difference between dirty superconductors and dirty Bose liquids. *Phys. Rev. B* **43**, 3740–3743 (1991).

2. J. A. van Dam, Y. V. Nazarov, E. P. A. M. Bakkers, L. P. Kouwenhoven, Supercurrent reversal in quantum dots. *Nature (London)* **442**, 667–670 (2006).

3. D. Razmadze, E. C. T. O'Farrell, P. Krogstrup, C. M. Marcus, Quantum dot parity effects in trivial and topological Josephson junctions. *Phys. Rev. Lett.* **125**, 116803 (2020).

4. A. Zazunov, R. Egger, T. Jonckheere, T. Martin, Anomalous Josephson current through a spin-orbit coupled quantum dot. *Phys. Rev. Lett.* **103**, 147004 (2009).

5. A. Brunetti, A. Zazunov, A. Kundu, R. Egger, Anomalous Josephson current, incipient time-reversal symmetry breaking, and Majorana bound states in interacting multilevel dots. *Phys. Rev. B* **88**, 144515 (2013).

6. D. B. Szombati, S. Nadj-Perge, D. Car, S. R. Plissard, E. P. A. M. Bakkers, L. P. Kouwenhoven, Josephson ϕ_0-junction in nanowire quantum dots. *Nat. Phys.* **12**, 568–572 (2016).

7. C. Schrade, S. Hoffman, D. Loss, Detecting topological superconductivity with φ_0 Josephson junctions. *Phys. Rev. B* **95**, 195421 (2017).

8. T. A. Fulton, P. L. Gammel, D. J. Bishop, L. N. Dunkleberger, G. J. Dolan, Observation of combined Josephson and charging effects in small tunnel junction circuits. *Phys. Rev. Lett.* **63**, 1307–1310 (1989).

9. L. J. Geerligs, V. F. Anderegg, J. Romijn, J. E. Mooij, Single Cooper-pair tunneling in small-capacitance junctions. *Phys. Rev. Lett.* **65**, 377–380 (1990).

10. M. T. Tuominen, J. M. Hergenrother, T. S. Tighe, M. Tinkham, Experimental evidence for parity-based 2*e* periodicity in a superconducting single-electron tunneling transistor. *Phys. Rev. Lett.* **69**, 1997–2000 (1992).

11. D. J. van Woerkom, A. Geresdi, L. P. Kouwenhoven, One minute parity lifetime of a NbTiN Cooper-pair transistor. *Nat. Phys.* **11**, 547–550 (2015).

12. J. van Veen, A. Proutski, T. Karzig, D. I. Pikulin, R. M. Lutchyn, J. Nygård, P. Krogstrup, A. Geresdi, L. P. Kouwenhoven, J. D. Watson, Magnetic-field-dependent quasiparticle dynamics of nanowire single-Cooper-pair transistors. *Phys. Rev. B* **98**, 174502 (2018).

13. A. Proutski, D. Laroche, B. Van 'T Hooft, P. Krogstrup, J. Nygård, L. P. Kouwenhoven, A. Geresdi, Broadband microwave spectroscopy of semiconductor nanowire-based Cooper-pair transistors. *Phys. Rev. B* **99**, 220504 (2019)

14. C. Schrade, L. Fu, Andreev or Majorana, Cooper finds out. arXiv:1809.06370 [cond-mat.mes-hall] (2018).

15. C. W. J. Beenakker, D. DiVincenzo, C. Emary, M. Kindermann, Charge detection enables free-electron quantum computation. *Phys. Rev. Lett.* **93**, 020501 (2004).

16. W. Mao, D. V. Averin, R. Ruskov, A. N. Korotkov, Mesoscopic quadratic quantum measurements. *Phys. Rev. Lett.* **93**, 056803 (2004).

17. H.-A. Engel, D. Loss, Fermionic Bell-state analyzer for spin qubits. *Science* **309**, 586–588 (2005).

18. K. Lalumière, J. M. Gambetta, A. Blais, Tunable joint measurements in the dispersive regime of cavity QED. *Phys. Rev. A* **81**, 040301 (2010).

19. R. Ionicioiu, Entangling spins by measuring charge: A parity-gate toolbox. *Phys. Rev. A* **75**, 032339 (2007).

20. W. Pfaff, T. H. Taminiau, L. Robledo, H. Bernien, M. L. Markham, D. J. Twitchen, R. Hanson, Demonstration of entanglement-by-measurement of solid-state qubits. *Nat. Phys.* **9**, 29–33 (2013).

21. C. K. Andersen, A. Remm, S. Balasiu, S. Krinner, J. Heinsoo, J.-C. Besse, M. Gabureac, A. Wallraff, C. Eichler, Entanglement stabilization using ancilla-based parity detection and real-time feedback in superconducting circuits. *npj Quantum Inf.* **5**, 69 (2019).

22. C. C. Bultink, T. E. O'Brien, R. Vollmer, N. Muthusubramanian, M. W. Beekman, M. A. Rol, X. Fu, B. Tarasinski, V. Ostroukh, B. Varbanov, A. Bruno, L. DiCarlo, Protecting quantum entanglement from leakage and qubit errors via repetitive parity measurements. *Sci. Adv.* **6**, eaay3050 (2020).

23. D. Aasen, M. Hell, R. V. Mishmash, A. Higginbotham, J. Danon, M. Leijnse, T. S. Jespersen, J. A. Folk, C. M. Marcus, K. Flensberg, J. Alicea, Milestones toward Majorana-based quantum computing. *Phys. Rev. X* **6**, 031016 (2016).

24. S. Plugge, A. Rasmussen, R. Egger, K. Flensberg, Majorana box qubits. *New J. Phys.* **19**, 012001 (2017).

25. T. Karzig, C. Knapp, R. M. Lutchyn, P. Bonderson, M. B. Hastings, C. Nayak, J. Alicea, K. Flensberg, S. Plugge, Y. Oreg, C. M. Marcus, M. H. Freedman, Scalable designs for quasiparticle-poisoning-protected topological quantum computation with Majorana zero modes. *Phys. Rev. B* **95**, 235305 (2017).

26. C. Schrade, L. Fu, Majorana superconducting qubit. *Phys. Rev. Lett.* **121**, 267002 (2018).

27. C. Schrade, L. Fu, Quantum Computing with Majorana Kramers Pairs. arXiv:1807.06620 [cond-mat.mes-hall] (2018).

28. S. Gazibegovich, D. Car, H. Zhang, S. C. Balk, J. A. Logan, M. W. A. de Moor, M. C. Cassidy, R. Schmits, D. Xu, G. Wang, P. Krogstrup, R. L. M. Op het Veld, J. Shen, D. Bouman, B. Shojaei, D. Pennachio, J. S. Lee, P. J. van Veldhoven, S. Koelling, M. A. Verheijen, L. P. Kouwenhoven, C. J. Palmstrøm, E. P. A. M. Bakkers, Epitaxy of advanced nanowire quantum devices. *Nature* **548**, 434–438 (2017).

29. S. M. Albrecht, A. P. Higginbotham, M. Madsen, F. Kuemmeth, T. S. Jespersen, J. Nygård, P. Krogstrup, C. M. Marcus, Exponential protection of zero modes in Majorana islands. *Nature* **531**, 206–209 (2016).

30. J. Shen, S. Heedt, F. Borsoi, B. van Heck, S. Gazibegovich, R. L. M. Op het Veldd, D. Car, J. A. Logan, M. Pendharkar, S. J. J. Ramakers, G. Wang, D. Xu, D. Bouman, A. Geresdi, C. J. Palmstrøm, E. P. A. M. Bakker, L. P. Kouwenhoven, Parity transitions in the superconducting ground state of hybrid InSb-Al Coulomb islands. *Nat. Commun.* **9**, 4801 (2018).

31. J. Shen, G. W. Winkler, F. Borsoi, S. Heedt, V. Levajac, J.-Y. Wang, D. van Driel, D. Bouman, S. Gazibegovic, R. L. M. Op Het Veld, D. Car, J. A. Logan, M. Pendharkar, C. J. Palmstrøm, E. P. A. M. Bakkers, L. P. Kouwenhoven, B. van Heck, Full parity phase diagram of a proximitized nanowire island. *Phys. Rev. B* **104**, 045422 (2021).

32. S. Vaitiekenas, M. T. Deng, J. Nygård, P. Krogstrup, C. M. Marcus, Effective *g* factor of subgap states in hybrid nanowires. *Phys. Rev. Lett.* **121**, 037703 (2018).

33. L. Bretheau, Localized Excitations in Superconducting Atomic Contacts: PROBING THE ANDREEV DOUBLET. Thesis, CEA-Saclay, Gif-sur-Yvette Cedex (2013).

34. V. Ambegaokar, B. I. Halperin, Voltage due to thermal noise in the dc Josephson effect. *Phys. Rev. Lett.* **22**, 1364–1366 (1969).

35. H. Ingerslev Jørgensen, T. Novotný, K. Grove-Rasmussen, K. Flensberg, P. E. Lindelof, Critical current 0–π transition in designed Josephson quantum dot junctions. *Nano Lett.* **7**, 2441–2445 (2007).

36. A. C. C. Drachmann, H. J. Suominen, M. Kjaergaard, B. Shojaei, C. J. Palmstrøm, C. M. Marcus, F. Nichele, Proximity effect transfer from NbTi into a semiconductor heterostructure via epitaxial aluminum. *Nano Lett.* **17**, 1200–1203 (2017).

37. M. Tinkham, *Introduction to Superconductivity* (Dover Publications, INC. ed. 2, 2004).

38. J. G. Kroll, Magnetic field compatible hybrid circuit quantum electrodynamics. Thesis, Delft University of Technology, Delft (2019).

39. A. J. Annunziata, D. F. Santavicca, L. Frunzio, G. Catelani, M. J. Rooks, A. Frydman, D. E. Prober, Tunable superconducting nanoinductors. *Nanotechnology* **21** 445202 (2010).
